# Patients with depression symptoms are more likely to experience improvements of internet-based cognitive behavioral therapy: a secondary analysis of effect modifiers in patients with non-cardiac chest pain in a randomized controlled trial

**DOI:** 10.1186/s12888-023-05238-1

**Published:** 2023-10-14

**Authors:** Terje Thesen, Joseph A. Himle, Are Hugo Pripp, Tor Sunde, Liv T. Walseth, Frode Thorup, Frode Gallefoss, Egil Jonsbu

**Affiliations:** 1 DPS Solvang, Sørlandet Hospital, SSHF, Servicebox 416, Kristiansand, 4604 Norway; 2 Faculty of Medicine and Health Science, Norwegian University of Science and Technology, Trondheim, Norway; 3 School of Social Work and School of Medicine-Psychiatry, University of Michigan, Ann Arbor, USA; 4 Oslo Centre of Biostatistics and Epidemiology, Research Support Services, Oslo University Hospital, Oslo, Norway; 5 Department of Clinical Research, Sørlandet Hospital, SSHF, Kristiansand, Norway; 6 Department of Cardiology, Sørlandet Hospital, SSHF, Kristiansand, Norway; 7 Faculty of Medicine, University of Bergen, Bergen, Norway; 8 Department of Psychiatry, Møre and Romsdal Hospital Trust, Molde, Norway

**Keywords:** Non-cardiac chest pain, Internet-based treatment, Internet-assisted treatment, iCBT, Cognitive behavioral therapy, Randomized controlled trial, Psychosomatic medicine

## Abstract

**Background:**

Non-cardiac chest pain is common and associated with increased anxiety and reduced health-related quality of life. Randomized controlled trials on psychological interventions for patients with non-cardiac chest pain have reported mixed results. Patients with non-cardiac chest pain are a heterogeneous group. Identifying sub-groups that could potentially benefit more (or less) from an intervention would be valuable knowledge. We have conducted a randomized controlled trial where internet-based cognitive behavioural therapy (iCBT) had effect on reducing cardiac anxiety and increasing health-related quality of life at 12-month follow-up. The aim of the present study was to explore potential effect modifiers of iCBT in patients with non-cardiac chest pain on cardiac anxiety and/or health related quality of life at 12-month follow-up.

**Methods:**

We analysed data from our randomized, controlled trial where 161 patients with non-cardiac chest pain were included and randomized to either iCBT or a treatment as usual (control). Cardiac anxiety measured by the Cardiac Anxiety Questionnaire and health-related quality of life measured by the EuroQol Visual Analog Scale at 12 month follow-up were the primary outcomes. Four potential baseline characteristics where identified as potential effect modifiers by a theory-based approach: (1) depression measured by the Patient Health Questionnaire; (2) anxiety measured by the Body Sensations Questionnaire; (3) prior healthcare contacts measured by a self-developed question; and (4) chest pain frequency measured by a self-developed question. Each potential effect modifier was analysed in a linear regression model where cardiac anxiety and EQ-VAS scores at 12-month follow-up, separately, were used as dependent variables. The potential differential treatment effect for each effect modifier was assessed by the interaction term: effect modifier x treatment group.

**Results:**

Depression symptoms at baseline predicted a differential treatment effect at 12-month follow-up on health-related quality of life in favor of the iCBT group (regression coefficient of the interaction term: -1.85 (CI -3.28 to -0.41), *p* = 0.01), but not on cardiac anxiety at 12-month follow-up. Fear of bodily symptoms, chest pain frequency and prior health care contacts at baseline did not predict a treatment effect on either health-related quality of life or cardiac anxiety.

**Conclusions:**

Depression symptoms at baseline predicted a positive treatment effect of iCBT on health-related quality of life in patients suffering from non-cardiac chest pain. This indicates that it is important to identify patients with non-cardiac chest pain and co-occurring depression symptoms given that they are particularly likely to benefit from iCBT.

**Trial registration:**

ClinicalTrials.gov NCT03096925.

## Introduction

More than half of patients presenting with chest pain at a hospital are discharged with a diagnosis of non-cardiac chest pain [1]. After excluding a cardiac origin of their chest pain, these patients are typically discharged without any specific follow-up. More than 40% of patients with non-cardiac chest pain have sustained complaints six months after a negative cardiac examination, including reduced quality of life, increased anxiety and avoidance of physical activity [2]. Due to high prevalence, increased healthcare utilization and indirect costs (e.g. sick leave), patients with non-cardiac chest pain generate significant costs to society [3]. In sum, patients with non-cardiac chest pain often experience enduring hardships, and the associated costs are high.

Previous studies have found that patients with non-cardiac chest pain have increased levels of psychological distress compared to a healthy population, and possibly worse than chest pain patients with a cardiac origin [4–6]. Anxiety in patients with non-cardiac chest pain is associated with a poor prognosis, reduced health-related quality of life and increased health care utilization [7]. Notably, panic disorder yields a particularly poor prognosis and is associated with reduced health-related quality of life in patients with non-cardiac chest pain [8]. Depression is also common among patients with non-cardiac chest pain and has been found to be associated with persistent complaints and reduced health-related quality of life [2, 7]. Thus, both anxiety and depression are important intervention targets for patients with non-cardiac chest pain.

There is no standard treatment for patients with non-cardiac chest pain. Although the results are mixed, psychological interventions are likely to be helpful in terms of reducing cardiac related anxiety, chest pain related complaints and in improving quality of life [9, 10]. A Cochrane review from 2015 concluded that cognitive behavioral therapy, delivered in a face-to-face format, had promising effects in the short term for non-cardiac chest pain [10]. Cognitive behavioral therapy aims at improving symptoms by identifying and modifying cognitive misperceptions and maladaptive behavior while promoting alternate, adaptive ways of coping with the symptoms. The Cochrane review pointed to several barriers to implementing cognitive behavioral therapy for non-cardiac chest pain including limited access to psychotherapists trained in the treatment of non-cardiac chest pain, low psychological attribution to non-cardiac chest pain symptoms and a high degree of heterogeneity among patients with non-cardiac chest pain [10]. There is a clear need for a treatment approach that overcomes these access barriers, and internet-based cognitive behavioral therapy (iCBT) is a promising, potential solution [11].

Since the Cochrane review (2015), a limited number of controlled trials with mixed results have investigated both face-to-face and iCBT treatments for non-cardiac chest pain [9, 12–17]. Overall, these studies provide continued evidence for a short-term (3-month) effect of cognitive behavioral therapy on non-cardiac chest pain, but long-term (12-month or more) follow-up studies remain rare.

Primary analysis from our previously published randomized controlled trial found positive effects of iCBT vs. ‘treatment as usual’ over the long term. The intervention was effective in reducing cardiac anxiety and improving health-related quality of life at 12-month follow-up [9]. Further, our study indicated that patients with higher pre-treatment levels of depression symptoms particularly profited from the intervention. Conversely, Mourad et al. (2022) recently reported 3-month follow-up data on iCBT compared to psychoeducation alone and found that iCBT was not superior to psychoeducation on reducing cardiac anxiety. However, they reported non-significant, long-term improvements on all variables, and notably, a trend indicating a similar moderate effect of iCBT on health-related quality of life as found in our study [17]. Thus, iCBT might be effective in reducing cardiac anxiety and particularly improving health-related quality of life.

Several studies have researched which variables are likely to predict a poor outcome in patients with non-cardiac chest pain; however, to our knowledge, only one study has investigated who is particularly likely to *benefit* from cognitive behavioral therapy [14]. Still, several cognitive behavioral intervention studies have made reasonable assumptions regarding who will be most likely to profit by only including patients with certain characteristics (e.g. above a certain anxiety level, with more than one healthcare contact regarding chest pain, above a certain chest pain frequency and/or significant complaints). As noted earlier, anxiety and depression predicts poor outcome in patients with non-cardiac chest pain, and our study suggested that those with co-occurring depressive symptoms at baseline might benefit more from our iCBT [2, 9, 18]. In addition, Mulder et al. (2019) tested brief face-to-face cognitive behavioral therapy on an unselected (i.e., included regardless of symptom level or prior health care visits) group of patients with non-cardiac chest pain, and found that those with at least one prior healthcare visit with non-cardiac chest pain had fewer at hospital attendances in the short term, but not in the long term [14]. Frequent chest pain is also a strong predictor of increased health care utilization [19] and is associated with lower general health [20]. Thus, one might hypothesize that anxiety, depression, prior healthcare visits and/or chest pain frequency may influence the effect of cognitive behavioral therapy for non-cardiac chest pain, but there is a need for further studies examining this issue.

Overall, patients with non-cardiac chest pain are a heterogeneous group and treatment results are mixed. Primary analysis from our previously published randomized controlled trial indicated that our brief, easy to distribute, low-resource utilizing, internet-based cognitive behavioral therapy intervention is helpful for unselected patients with non-cardiac chest pain, suggesting that it could be useful to offer the intervention to everyone who presents for evaluation of chest pain that turns out to be of non-cardiac origin [9]. However, it is important to note that these patients are heterogeneous with broad variation in symptoms, needs and outcomes [10]. In addition, participation rates in studies are often low, possibly due to the fact that many patients with non-cardiac chest pain may not attribute their pain to a psychological cause [10]. It is therefore likely that some patients in need of the treatment, may refuse it, or some may accept it but not benefit from it. Extending the research on our internet-based cognitive therapy intervention to include possible effect modifiers is especially important to identify which patients are most likely to benefit from it. We wanted in this study to investigate whether depression, anxiety, prior healthcare contacts and/or chest pain frequency modify the effect of internet-based treatment for patients with non-cardiac chest pain on measures of cardiac anxiety and/or health-related quality of life.

## Methods

We hypothesized that one or more of four patient characteristics (described below) could modify the effect of internet-based cognitive behavioral therapy (iCBT) on cardiac anxiety and/or health-related quality of life in patients with non-cardiac chest pain at 12-month follow-up.

### Design

Our analysis in the present paper on effect modifiers is based on data from our non-cardiac chest pain study, a randomized controlled trial with a 12-month follow-up period. The intervention group received internet-based cognitive behavioral therapy and the control group received usual care. Detailed study methods and primary analysis have been described previously [9].

### Participants and recruitment

We recruited participants at Sørlandet Hospital, Kristiansand, Norway from April 2017 to March 2018. Eligible patients were referred with chest pain as their main complaint to the cardiac department, and had no cardiac or obvious somatic explanation for their chest pain, and no history of, or current, severe heart disease. Patients with language difficulties, no access to internet, obvious cognitive impairment, severe comorbidities and/or inability to do physical activity were excluded. All participants gave informed consent and signed a written consent form, and the study was approved by the Regional Committee for Medical Research Ethics (2014/2031). Details of the recruitment procedure and description of the intervention have previously been described [9, 21].

### Control group

Participants in the control group received usual care. Usual care, for most participants, was comprised of a personal consultation with a cardiac specialist after a complete cardiac examination, which included coronary computer tomography for nearly all participants.

### Intervention group

The intervention group received, in addition to usual care, the first session of the iCBT immediately after initial consultation with the cardiac specialist. The iCBT had six sessions that the participant completed autonomously. The aim of the iCBT was to enhance mastery of non-cardiac chest pain by psychoeducation, cognitive behavioral therapy elements (e.g., explain the relationship between stress/anxiety and bodily symptoms, provide an alternate explanation for their chest pain instead of heart disease, discuss behavior that can maintain the problem and encourage exposure to physical activity) and physical activity. The participants had weekly brief telephone support in between sessions where the participants planned, performed, and reported tailored physical activity tasks aimed at giving participants the experience that physical activity is safe. The intervention has been previously described in detail [9, 21].

### Choice of variables

The choice of potential effect modifiers were theory-based as recommended by Heinze et al. [22], however, they were not predefined in ClinicalTrials.gov. We previously found that the iCBT was effective in reducing cardiac anxiety and increasing health-related quality of life at 12-month follow-up in patients with non-cardiac chest pain. In that study, 12-month follow-up was chosen as the primary endpoint because the long-term effect of the intervention was regarded as particularly important. Thus, we chose cardiac anxiety and health-related quality of life at 12-month follow-up as the intervention effects of interest.

Based on a review of the literature on non-cardiac chest pain as well as experience from the present study, the research group identified three domains that could potentially influence the effect of the iCBT:

1. Psychological factors
2. Utilization of health care services
3. Chest pain characteristics

### Psychological factors

We chose depression and anxiety symptoms measured by the Patient Health Questionnaire (PHQ-9) and Bodily Sensations Questionnaire (BSQ) as possible effect modifiers from the psychological domain.

Depression symptoms at baseline predicts poor outcome and a reduced health-related quality of life in patients with non-cardiac chest pain [2, 7]. In our study we previously found that participants with PHQ-9 score ≥ 5 seemed to have a stronger effect of the intervention, however, this was not analyzed further [9]. This suggest that patients with non-cardiac chest pain and depressive symptoms might have a differential treatment effect of iCBT. To our knowledge, no earlier studies have investigated depression symptoms as an effect modifier in treatments of non-cardiac chest pain.

Higher scores of BSQ are associated with anxiety and particularly panic symptoms [23]. Cut off scores of BSQ have also been used as one of the inclusion criteria in a prior non-cardiac chest pain study [13]. It seems reasonable to assume that participants with higher levels of anxiety will be more likely to benefit from a non-cardiac chest pain intervention because most of these interventions target anxiety specifically. However, to our knowledge this has not been formally investigated.

### Health care utilization

Patients with non-cardiac chest pain utilize a significant portion of available health care services [3]. More than one prior hospital visit has been used as an inclusion criterion in earlier iCBT studies targeting non-cardiac chest pain [13, 17]. Another study indicated that patients with non-cardiac chest pain and more than one prior contact with the health care system related to non-cardiac chest pain seemed to benefit more from a brief face-to-face non-cardiac chest pain intervention in the short term [14]. Thus, we have included the investigator developed baseline question *“Have you been in contact with the healthcare system during the last year because of chest pain besides the current episode? (If yes, what kind of contact)”* as a potential effect modifier.

### Chest pain characteristics

Frequency of non-cardiac chest pain has been reported as a strong predictor for health care visits [19]. Increased utilization of health care services in patients with non-cardiac chest pain might indicate an increased burden of disease, and those patients might be in a greater need of and might profit more from iCBT. We have therefore included the investigator developed baseline question *“How often do you experience chest pain”* with the following possible responses: (1) daily, (2) weekly, (3) sometimes, (4) never, as a possible effect modifier. We have made the variable dichotomous for the analyses; (1) and (2) are put together as well as (3) and (4).

### Assessments

We assessed participants at baseline, post treatment, and at 3- and 12-month follow-up. Demographic and clinical data were collected at baseline as reported in Table 1. We used the following assessments:

**Table 1:** Demographic data and possible effect modifiers collected at baseline

	iCBT group, *n* = 80	Treatment as usual, *n* = 81	Total sample, *n* = 161
Women (%)	44 (55)	43 (53)	87 (54)
Age, median (range)	53 (20–69)	51 (30–69)	52 (20–69)
**Possible effect modifiers:**			
**PHQ-9 (SD)**	6.9 (4.7)	6.9 (5.0)	6.9 (4.8)
**BSQ (SD)**	36.8 (11.6)	36.7 (12.9)	36.7 (12.2)
**Health care contacts (SD)**			
Yes (%)	49 (61)	36 (44)	85 (53)
No (%)	31 (39)	45 (56)	76 (47)
**Chest pain frequency**			
Never/Seldom (%)	43 (54)	41 (51)	84 (52)
Weekly/Daily (%)	37 (46)	40 (49)	77 (48)

#### Cardiac Anxiety Questionnaire (CAQ)

The CAQ measures cardiac anxiety. It consists of 18 items scored on a scale from 0–4, and yields a total score ranging from 0 (no symptoms) to 72 (extreme symptoms) [24]. The CAQ is validated, has adequate internal consistency (Cronbach α = 0.84) and good test–retest reliability (*r* = 0.88) [25, 26].

#### EuroQol Visual Analog Scale (EQ-VAS)

The EQ-VAS measures self-reported health related quality of life using a visual analog scale from 0 to 100, where 0 is defined as the worst imaginable state of health, and 100 is the best imaginable state of health [27]. The EQ-VAS is an integral part of the EQ-5D, which is a widely used and validated self-report questionnaire to assess health-related quality of life. The first part of the EQ-5D consists of questions regarding problem severity in five different health domains (i.e., mobility, self-care, usual activities, pain/discomfort and anxiety/depression), whereas the last part of the EQ-5D is the EQ-VAS. The first part of the EQ-5D represents the societal perspective suitable for health economic analysis whereas the last part (EQ-VAS) represents the participant’s perspective suitable for a clinical assessment [28]. In the present study we chose to report on the EQ-VAS, while the first part of the EQ-5D will be used in a later health economic analysis.

#### Patient Health Questionnaire (PHQ-9)

The PHQ measures depression symptoms. It consists of nine items scored on a scale from 0 to 3, and a sum score range of 0 (no symptoms) to 27 (severe symptoms) [29]. A score of 5 to 9 indicates mild depressive symptoms, 10 to 14 mild to moderate depressive symptoms, 15 to 19 moderate depressive symptoms and ≥ 20 severe depressive symptoms. The PHQ-9 is validated, has good internal consistency (Cronbach α = 0.89) and good test–retest reliability (*r* = 0.84) [29].

#### Body Sensations Questionnaire (BSQ)

The BSQ measures fear/worry about different bodily sensations. It consists of 17 items scored on a scale from 1 to 5, and a sum score range of 17 (no symptoms) to 85 (extreme symptoms) [23]. The BSQ is validated, has an adequate internal consistency (Cronbach α = 0.87) and moderate test–retest reliability (*r* = 0.66) [23, 30].

### Statistical analysis

The Statistical Analysis Plan for this study was determined after the database was opened, but before the statistical tests were performed. We used software package Stata version SE 17 for all analyses.

We analyzed the effect of each of the potential effect modifiers with a linear regression model where we used cardiac anxiety and EQ-VAS scores at 12-month follow-up, separately, as dependent variables. Independent variables were treatment group, cardiac anxiety or EQ-VAS score at baseline, and the interaction term effect modifier x treatment group. The difference of effect across the levels of each possible effect modifier was assessed by the interaction term. Mean differences for cardiac anxiety and EQ-VAS at 12-month follow-up between the control group and iCBT group for each effect modifier were reported by pairwise comparison of margins in the model by the interaction term for the categorical effect modifiers (health care contacts and chest pain frequency). If the test shows that the interaction term is statistically significant, it indicates that the observed difference in treatment effect between the subgroups (e.g. high vs low frequency of chest pain) is because of the effect modifier (e.g. frequency of chest pain) in the model.

The power was estimated for the main effects and not for sub-group analysis. Since the sample had a lower power than needed to be sure to detect a difference, we focused not only on statistical significance, but also on direction of the effects. Because the analyses were meant to be hypothesis generating, adjustments for multiple testing were not performed.

## Results

Flow of participants has been previously reported [9]. Out of 231 invited patients, 162 (70.1%) accepted participation and were randomized 1:1 to either the control group (*n* = 81) or the internet-based cognitive behavioral therapy (iCBT) group (*n* = 81). The iCBT sessions (at least 5 out of 6) were completed by 68 participants (84%). Assessments at 12-month follow-up were completed by 56 (69%) in the iCBT group and 61 (75%) in the control group.

Demographic data and baseline factors according to iCBT and control group are presented in Table 1.

### Analysis of possible effect modifiers

#### Depression symptoms measured by PHQ-9

**Fig. 1:**
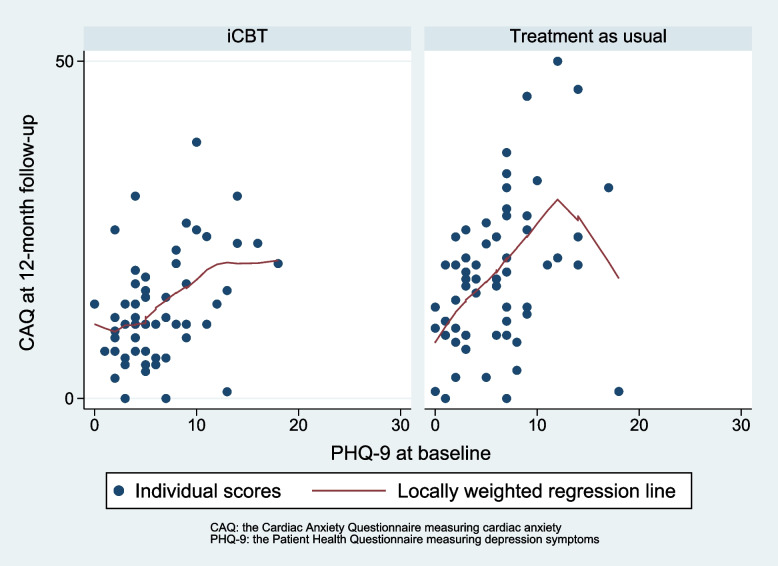
Relationship between depression scores at baseline and cardiac anxiety at 12-month follow-up for the intervention group and treatment as usual group

The analysis found no effect modification of depression level at baseline on cardiac anxiety at 12-month follow-up, as illustrated in Fig. 1.

The interaction term group x PHQ score at baseline shows a statistically non-significant regression coefficient of 0.25 (CI -0.38 to 0.88), *p* = 0.44 on CAQ at 12-month follow-up. In Fig. 1, the regression lines show a tendency towards that increasing PHQ-9 score at baseline had a less negative effect on CAQ at 12-month follow-up in the iCBT group compared to the control group. Thus, depression symptoms might modify the effect of treatment.

**Fig. 2:**
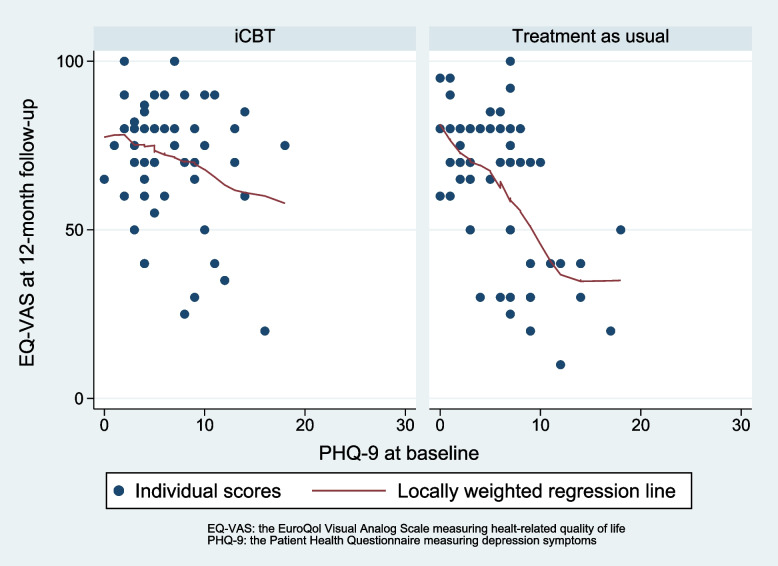
Relationship between depression scores at baseline and health-related quality of life at 12-month follow-up for the intervention group and treatment as usual group

The analysis found an effect modification of depression level at baseline on health-related quality of life at 12-month follow-up, as illustrated in Fig. 2.

The interaction term group x PHQ score at baseline shows a statistically significant regression coefficient of -1.85 (CI -3.28 to -0.41), *p* = 0.01 on EQ-VAS at 12-month follow-up. Lower number indicate worse health-related quality of life. In Fig. 2, the regression lines show that increasing PHQ-9 score at baseline had a clearly less negative effect on EQ-VAS at 12-month follow-up in the iCBT group compared to the control group (i.e., effect modification between depression symptoms and treatment groups).

#### Fear of bodily sensations measured by BSQ

**Fig. 3:**
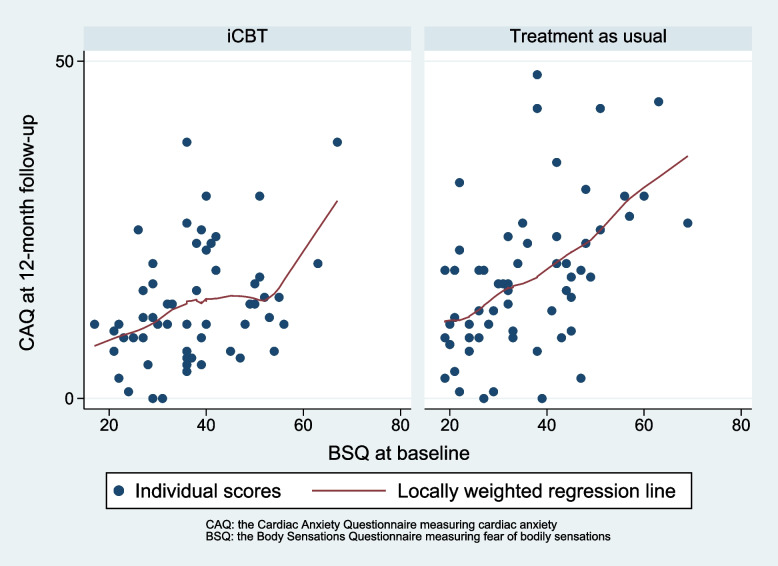
Relationship between fear of bodily sensations at baseline and cardiac anxiety at 12-month follow-up for the intervention group and treatment as usual group

The analysis found no effect modification of fear of bodily sensations level at baseline on cardiac anxiety at 12-month follow-up, as illustrated in Fig. 3.

The interaction term group x BSQ score at baseline shows a statistically non-significant regression coefficient of 0.11 (CI -0.12 to 0.33), *p* = 0.35 on CAQ at 12-month follow-up. In Fig. 3, the regression lines show a tendency towards that increasing BSQ score at baseline had a less negative effect on CAQ at 12-month follow-up in the iCBT group compared to the control group.

**Fig. 4:**
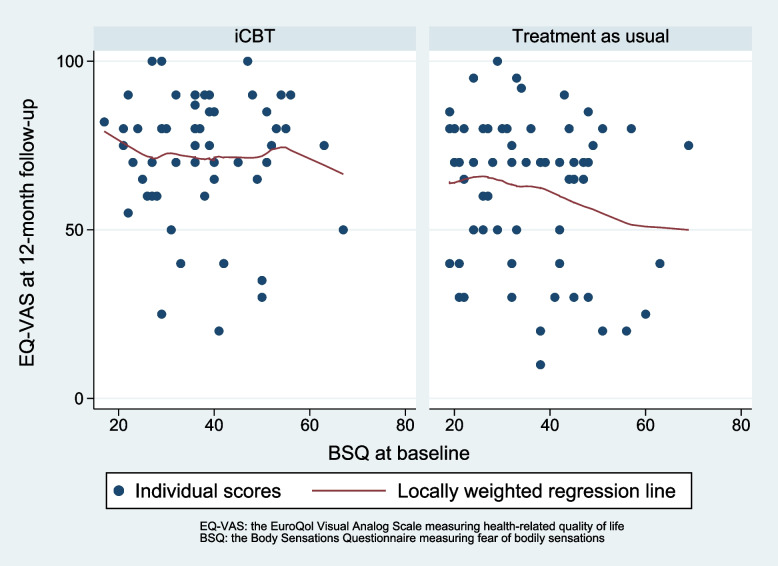
Relationship between fear of bodily sensation scores at baseline and health-related quality of life at 12-month follow-up for the intervention group and treatment as usual group

The analysis found no effect modification of fear of bodily sensations level at baseline on health-related quality of life at 12-month follow-up, as illustrated in Fig. 4.

The interaction term group x BSQ score at baseline shows a statistically non-significant regression coefficient of 0.076 (CI -0.47 to 0.62), *p* = 0.78 on EQ-VAS at 12-month follow-up. In Fig. 4, the regression lines show a tendency towards that increasing BSQ score at baseline had a less negative effect on EQ-VAS at 12-month follow-up in the iCBT group compared to the control group.

#### Health care contacts because of chest pain in the last 12 months (yes or no)

The analysis found no effect modification of prior health contacts on either cardiac anxiety at 12-month-follow-up or health-related quality of life at 12-month follow-up.

The difference between the iCBT group and control group on CAQ at 12-month follow-up for those with prior health care contacts was 1.83 (CI -1.96 to 5.61), *p* = 0.34, and the difference between the iCBT group and control group for those without prior health care contacts on CAQ at 12-month follow-up was 5.78 (CI 1.92 to 9.68), *p* = 0.004. The interaction term group x health care contacts at baseline shows a statistically non-significant regression coefficient of 3.97 (CI -1.46 to 9.41), *p* = 0.15. The direction of the effect on CAQ in the iCBT group was in favor of those with no former health care contacts in the last 12 months at baseline, opposite to our a priori hypothesis.

The difference between the iCBT group and control group for those with prior health care contacts on EQ-VAS at 12-month follow-up was 14.2 (CI 23.1 to 5.3), *p* = 0.002, and the difference between the iCBT group and control group for those without prior health care contacts on EQ-VAS at 12-month follow-up was 5.93 (CI 15.1 to -3.21), *p* = 0.20. The interaction term group x health care contacts at baseline shows a statistically non-significant regression coefficient of 8.31 (CI -4.48 to 21.1), *p* = 0.20. The direction of the effect on EQ-VAS in the iCBT group was in favor of those with prior health care contacts in the last 12 months at baseline.

#### Chest pain frequency

The analysis found no effect modification of chest pain frequency at baseline on either cardiac anxiety at 12-month-follow-up or health-related quality of life at 12-month follow-up.

The difference between the iCBT group and control group on CAQ at 12-month follow-up for those with low chest pain frequency was 2.06 (CI -1.45 to 5.57), *p* = 0.25, and the difference between the iCBT group and control group for those with high chest pain frequency on CAQ at 12-month follow-up was 5.65 (CI 1.65 to 9.64), *p* = 0.006. The interaction term group x chest pain frequency at baseline shows a statistically non-significant regression coefficient of 3.59 (CI -1.73 to 8.91), *p* = 0.18. The direction of the effect on CAQ in the iCBT group was in favor of those with high chest pain frequency at baseline.

The difference between the iCBT group and control group on EQ-VAS at 12-month follow-up for those with low chest pain frequency was -6.00 (CI -14.2 to 2.21), *p* = 0.15, and the difference between the iCBT group and control group for those with high chest pain frequency on EQ-VAS at 12-month follow-up was -16.0 (CI -25.3 to -6.65), *p* = 0.001. The interaction term group x chest pain frequency at baseline shows a statistically non-significant regression coefficient of -10.0 (CI -22.5 to 2.47), *p* = 0.12. The direction of the effect on EQ-VAS in the iCBT group was in favor of those with high chest pain frequency at baseline.

## Discussion

The present study aimed to explore if any of four potential effect modifiers (depression symptoms, fear of bodily symptoms, prior healthcare contacts because of chest pain, or chest pain frequency) influenced the effect of our internet-based cognitive behavioral therapy (iCBT) on cardiac anxiety or health-related quality of life for patients with non-cardiac chest pain. Patients with non-cardiac chest pain are a heterogeneous group and it would be beneficial to be able to identify those who are most likely to benefit from iCBT.

Depression symptoms at baseline predicted a differential treatment effect at 12-month follow-up on health-related quality of life in favor of the iCBT group, but not on cardiac anxiety. Fear of bodily symptoms, chest pain frequency and prior health care contacts at baseline did not predict a treatment effect on health-related quality of life or cardiac anxiety.

The difference in health-related quality of life between the control group and iCBT group at 12-month follow-up among participants with depressive symptoms at baseline indicates that depression is an important effect modifier on health-related quality of life. This finding was in line with our expectations, based on our prior published analysis [9] that indicated an enhanced treatment effect on patients with depression scores ≥ 5 (PHQ-9) at baseline. Furthermore, patients with non-cardiac chest pain who scored higher on baseline depressive symptoms profited more from iCBT compared to those with lower baseline depression symptoms. This finding it is a bit surprising because one could assume that patients with more depression symptoms would be in need of more extensive psychotherapeutic treatment beyond the brief internet-based approach used in this study.

Depression symptoms at baseline did not modify the effect on cardiac anxiety at 12-month follow-up. This finding was somewhat surprising, particularly since depression symptoms modified the effect on health-related quality of life at 12-month follow-up in favor of those assigned to iCBT. The reasons for the lack of an effect modification on cardiac anxiety is hard to determine. In our study almost all participants had been examined with a coronary computed tomography angiography which can rule out heart disease with a greater certainty than examinations that were more typically used some years ago (e.g., cardiac stress test). After the examination, the participants in most cases got a session with a cardiologist that included review of the results of the angiography which likely reassured them further. In addition, the cardiologist usually encouraged increased physical activity and we believe that this encouragement of physical activity might have introduced bias because the cardiologist knew about this focus in the intervention, and physical activity alone is probably helpful in reducing cardiac anxiety [31]. However, one important aspect in our study was that even though cardiac anxiety was improved significantly more in the iCBT group compared to the control group, both groups improved over time to a cardiac anxiety level similar to that of a normal age adjusted population [32]. The degree of improvement in cardiac anxiety in the control group was somewhat surprising given that other studies have noted poor psychological long-term outcomes in patients with non-cardiac chest pain [2, 8]. This supports the idea that there were some unknown factors, such as the examination method or cardiology session after the examination, that reduced cardiac anxiety in both groups. It is possible that cardiac anxiety was well enough treated in most patients by usual care alone in our setting, and in that case finding a differential treatment response on cardiac anxiety would be difficult.

To our knowledge, no previous studies have investigated depression symptoms as a potential effect modifier in patients with non-cardiac chest pain. However, previous studies have found that depression is a predictor of poor outcome in patients with non-cardiac chest pain [2]. Interestingly, there is a possible link between depression and heart disease. The presence of depression seems to increase the risk of developing heart disease, and the presence of depression in those with heart disease is a powerful predictor for worse outcomes in both reduced quality of life and premature death [33, 34]. Overall, this indicates that depression is a particularly important factor to pay attention to when persons present with non-cardiac chest pain. Our present results indicate that patients with non-cardiac chest pain should be screened for the presence of depression symptoms in order to identify patients that are most likely to profit from iCBT.

Fear of bodily symptoms (BSQ) did not predict a differential treatment effect of iCBT on either health-related quality of life or cardiac anxiety at 12-month follow-up. As stated earlier, BSQ is associated with anxiety and particularly panic symptoms. Previous studies have found that patients with non-cardiac chest pain and co-occurring panic symptoms have a particularly poor prognosis and often experience reduced quality of life [35]. Because our iCBT particularly targets anxiety, we anticipated that patients with higher levels of fear of bodily symptoms would respond particularly well to the intervention. However, this was not found. Based on the graph presenting the treatment effect at 12-month for different baseline levels of BSQ, it might be that those with moderate BSQ levels are more likely to profit from iCBT. However, the present sample size of participants with moderate baseline BSQ scores is small. If this hypothesis were true, it would imply that those with a high level of anxiety, as measured by BSQ, need a more comprehensive intervention than the present one to improve fears of bodily sensations. In our previously published results, our iCBT had a short-term, but not a long-term effect on improving BSQ [9]. We designed the iCBT based on Jonsbu et al.’s (2011) brief face-to-face cognitive behavioral therapy, which yielded a statistically and clinically significant long-term positive effect on BSQ compared to a treatment as usual control condition [36]. One difference from our study was that in one of Jonsbu et al.’s sessions the participants were exposed to maximum heart rate on a treadmill with the therapist present. This approach might have resulted in a particularly positive effect on those patients with higher fear of bodily symptoms because they are likely to have been previously reluctant to expose themselves to physical activity. Thus, exposure to vigorous physical activity with a therapist present could have reduced concerns about engaging in physical activity alone.

Previous healthcare contacts in the last 12-months due to chest pain did not predict a treatment effect on health-related quality of life or cardiac anxiety at 12-month follow-up. This result expands the findings by Mulder et al. (2019) testing brief face-to-face cognitive behavioral therapy for non-cardiac chest pain in a large randomized controlled trial [14]. They found that participants in the intervention group with at least one prior healthcare contact due to non-cardiac chest pain had less health care contacts in the short-term (3-month), but not in the long-term (12-month) [14], indicating that those with prior healthcare contacts could benefit more from an intervention. Other intervention studies have only included patients with non-cardiac chest pain with prior health care contacts due to non-cardiac chest pain, presumably because it was believed that those patients would profit more from the intervention under investigation. However, this is not supported by our findings and future studies should consider including patients with non-cardiac chest pain regardless of prior health care contacts.

Finally, chest pain frequency did not predict treatment effect on health-related quality of life or cardiac anxiety at 12-month follow-up. We assumed that those patients with non-cardiac chest pain with frequent non-cardiac chest pain episodes could potentially benefit more from the iCBT. A previous study found that chest pain frequency was a strong predictor of health care visits, indicating that these patients struggled more and therefore had greater potential to improve with iCBT [19]. We did not find a statistically significant difference in favor of the intervention group for those with a higher chest pain frequency, but there was a potential trend. In later studies it could be interesting to investigate chest pain frequency further as a possible predictor of treatment effect in a larger sample.

There are several limitations in this study. The potential effect modifiers were not predefined in the protocol at clinicaltrials.gov and they were determined after the database was opened. Severity and/or impact of non-cardiac chest pain were not included as a potential effect modifiers, and these could have been interesting factors because cardiac distress extends beyond anxiety, depression and chest pain frequency [37]. We calculated power in the original trial based on primary outcomes for the whole group, and not to detect differences in subgroups. This leads to a higher risk of a type II error, due to under powering. Because of the exploratory nature of this study, we did not adjust the significance level due to multiple testing, thus increasing the risk of a type I error. Given these factors, our results must be interpreted with caution and further studies are needed to increase knowledge on possible effect modifiers that influence treatment effects for persons receiving iCBT for non-cardiac chest pain.

These weaknesses notwithstanding, the study has several strengths. Key strengths are the randomized controlled design of the study and, although not pre-specified in the protocol, the potential effect modifiers we chose to analyze were theory-based and decided on prior to conducting the analysis. In addition, we included all patients with non-cardiac chest pain regardless of symptom level, prior hospital contacts, or non-cardiac chest pain frequency. Thus, our unselected group of participants forms a good base for exploring factors that might predict treatment response.

The present study has several potentially important clinical implications. We found that depression symptoms at baseline predicted a positive treatment effect of our internet-based cognitive behavioral intervention on health-related quality of life in patients suffering from non-cardiac chest pain. It has previously been reported that depression symptoms at baseline predicts a poor outcome in patients with non-cardiac chest pain [2]. This indicates that it is important to identify patients with non-cardiac chest pain and co-occurring depressive symptoms given that they are particularly likely to benefit from iCBT. We suggest that future research on non-cardiac chest pain interventions include depression symptoms as a potential effect modifier when studying the effect of non-cardiac chest pain interventions. If our findings are replicated, it is important that patients with non-cardiac chest pain are screened for depression when they present for care. This practice will reveal those patients who are most likely to profit from a low-cost, easily distributed internet-based cognitive behavioral intervention that can improve quality of life for persons with non-cardiac chest pain.

## Data Availability

The datasets used and analyzed during the current study are not publicly available due to data sharing restrictions by the Regional Committee for Medical Research Ethics, but are available from the corresponding author on reasonable request and if sharing of data is approved by the Regional Committee for Medical Research Ethics.

## Abbreviations

iCBT: Internet-based cognitive behavioural therapy
CAQ: Cardiac Anxiety Questionnaire
BSQ: Body Sensations Questionnaire
PHQ-9: Patient Health Questionnaire-9
EQ-VAS: EuroQol Visual Analog Scale
CI: Confidence interval
